# Nurses’ perceptions of family presence during resuscitation/CPR in emergency departments in Palestine: a cross-sectional study

**DOI:** 10.1186/s12912-026-05052-6

**Published:** 2026-07-18

**Authors:** Adam Marawaa, Yahia Abbad, Mohammad Hawamdah, Rebhi Bsharat, Hamdallah Khalid, Baraa Jammal, Mohammad Sultan, Ahmad Alhattawi

**Affiliations:** Department of Nursing, Modern University College, Ramallah, Palestine

**Keywords:** Family presence during resuscitation, Family-witnessed resuscitation, Emergency nursing, Cardiopulmonary resuscitation, Nurses’ attitudes, Palestine

## Abstract

**Background:**

In this study, the perception of nurses towards the presence of the family during the cardiopulmonary resuscitation (CPR) procedure in the Palestinian emergency room was investigated. Family involvement is increasingly encouraged to support the patient emotionally, although it creates some concerns.

**Methods:**

A descriptive cross-sectional design was employed in the West Bank to collect data from 150 nurses working in governmental and non-governmental institutions. Data collection was done using a systematic questionnaire. It included demographic data and perceptions about the presence of family during CPR.

**Results:**

The study examined opinions on family attendance during CPR in Palestinian EDs, consisting of 150 participants. Positive perception (> 2.1) was indicated by a total mean perception score of 2.22 (SD = 0.579). Participants were found to have a positive opinion on staff knowledge and family attendance in terminally ill patients, however, concerns were reported regarding patient comfort, appropriateness of family presence during resuscitation, and perceived benefits. No significant differences in perception were found in relation to years of experience, gender, age, occupation, or educational level. However, a significant difference in perception was found in relation to type of institution, with a p-value of 0.022, where participants from non-governmental hospitals scored higher than those from governmental hospitals, with a mean of 2.42 (SD = 0.67) and 2.17 (SD = 0.54), respectively.

**Conclusion:**

The study emphasizes the need for protocols, training, and cultural sensitivity in balancing the effectiveness of CPR with family-centered care. Resolution of these issues can help in improving attitudes and integrating family presence in CPR.

## Background

One of the most important life-saving techniques in emergency treatment, cardiopulmonary resuscitation (CPR), has become more and more successful globally, thanks to training programs and public awareness. It has been proven that CPR training for medical practitioners and the public, and educating the public about CPR, increases survival rates in cases of cardiac arrest [1].

One of the important and debatable facets of patient-centered care is the provision of family presence during resuscitation (FPDR). Healthcare providers have differing opinions about the provision of FPDR, even though professional guidelines have supported its provision as an option depending on institutional regulations. Studies have shown that although the process of watching resuscitation is stressful for the families, most of them prefer to be present and may have positive psychological outcomes and reduced anxiety if FPDR is provided [2].

The opinions of healthcare professionals on FPDR, however, are still unclear and sometimes conflicting. To identify obstacles and facilitate FPDR implementation in emergency department, it is crucial to comprehend their attitudes [3].

## Introduction

Family presence during resuscitation (FPDR) has emerged as a key aspect of patient-centered care on a worldwide level. Family presence during resuscitation can improve psychological outcomes and satisfaction, as highlighted by studies carried out in a number of countries, although opinions on FPDR are divided among medical experts worldwide. Although FPDR is supported by worldwide standards, its implementation varies significantly due to staff perspectives, institutional guidelines, and cultural issues. To effectively support FPDR in emergency care, it is imperative to comprehend these dynamics [4].

Moreover, cultural, ethical, and organizational issues influence the acceptability of FPDR, which in turn causes significant regional variability. According to some studies, family members present during the procedure often report emotional benefits in addition to a better understanding of medical care, though health professionals report challenges in managing high levels of stress, patient safety, and institutional challenges. The aforementioned studies highlight the need for developing specific policies and educational programs that address the needs of family members as well as the views of health professionals in order to facilitate the effective implementation of FPDR [5].

### Aims

Examine and evaluate nurses’ perceptions of family presence during resuscitation (CPR) in emergency departments.

## Methods

### Study setting and sample

A descriptive cross-sectional study was conducted to examine nurses’ perceptions toward the presence of family members during resuscitation/CPR in emergency departments in Palestine. The study was carried out in governmental and non-governmental hospitals across the West Bank, Palestine, between January and March 25, 2026.

Participants had to be staff nurses who worked in emergency rooms and provided direct patient care. Nurses who did not give informed consent, were not employed in emergency rooms, or were on leave at the time of data collection were not included.

A sample size calculator with a 95% confidence level and a ± 5% margin of error was used to determine the sample size, which resulted in a minimum required sample of 242 individuals. The sample size was increased by 10% to account for possible non-response. Participants were recruited using convenience sampling.

### Data collection tools and methods

A structured questionnaire created by [6]. was used to gather data. The questionnaire was adapted to fit the study context of family presence during resuscitation/CPR in emergency departments. It consisted of a ten-item scale measuring nurses’ perceptions regarding advantages and barriers of family presence. Responses were measured using a 3-point Likert scale ranging from 1 (disagree) to 3 (agree). The total perception score was calculated by summing all items, with higher scores indicating more positive perceptions.

There were two parts to the instrument. Demographic details including gender, age, highest level of education, years of experience, and years spent working in the emergency room were included in the first section. A ten-item attitude measure measuring nurses’ opinions about family presence during resuscitation, including perceived advantages and obstacles, was added in the second segment.

Participants’ perceptions were categorized using a predefined cut-off point. A mean score of less than 2.1 indicated negative perception, while a score of 2.1 or higher indicated positive perception, This cut-off was based on the midpoint of the 3-point Likert scale used in the questionnaire, where scores reflect the tendency toward disagreement or agreement.

The content validity was achieved by getting a panel of nursing experts review the questions and make recommendations; these were incorporated into the questionnaire. The reliability analysis showed acceptable internal consistency reliability; the results showed a coefficient alpha of 0.71. The questionnaires were administered to qualified nurses once approval was obtained from the Ministry of Health. Consent was obtained; however, the objective of the research was clearly explained, and participation in the research remained optional. Anonymity and confidentiality were observed during the data collection process.

### Data analysis

IBM SPSS version 27 software was used for data analysis. Descriptive statistics such as frequency, percentage, mean, and standard deviation were used to summarize the data. Normality tests such as Shapiro-Wilk and Kolmogorov-Smirnov tests were conducted to assess the distribution of the data. The homogeneity of variance was examined using Levene’s test. The independent t-test and one-way analysis of variance (ANOVA) were conducted to assess differences between variables. A p-value of less than 0.05 was considered statistically significant.

### Ethical approval

The participation was voluntary, and confidentiality was ensured by not using any identifying information. The participants were made aware of their right to withdraw at any stage without any penalty. The data was used for research purposes only.

## Results

The demographic characteristics of the study participants (*n* = 150) reveal that 69.3% were male and 30.7% were female. Regarding workplace distribution, 44.0% were from Northern Palestine (Jenin, Nablus, Tulkarm, Tubas, Qalqilya), 22.7% from Central Palestine (Jerusalem, Ramallah and Al-Bireh, Jericho and the Jordan Valley), and 33.3% from Southern Palestine (Hebron, Bethlehem).

Most participants, 75.3%, worked in government hospitals, while 24.7% were employed in non-governmental hospitals. In terms of age, 15.3% were aged 18–24 years, 49.4% were 25–34 years, 28.0% were 35–44 years, and 7.3% were 45–54 years. Educationally, 61.3% held a bachelor’s degree, 22.0% had higher education (master’s or PhD), and 16.7% held a diploma. Professional experience varied, with 34.7% having 1–5 years, 24.7% with 6–10 years, 20.7% with 11–15 years, 9.3% with 16–20 years, and 5.3% each with less than 1 year or more than 20 years. In the emergency department (ED) setting, 46.7% had 1–5 years of experience, 24.7% had less than 1 year, 19.3% had 6–10 years, and 9.3% had over 10 years.

The demographic characteristics of the participants are presented in Table 1.

**Table 1:** Demographic variables of the participants (*n* = 150)

Demographic variables		*n*	%
Sex	Male	104	69.3%
	Female	46	30.7%
Workplace	Northern Palestine (Jenin, Nablus, Tulkarm, Tubas, Qalqilya)	66	44.0%
	Central Palestine (Jerusalem, Ramallah and Al-Bireh, Jericho and the Jordan Valley)	34	22.7%
	Southern Palestine (Hebron, Bethlehem)	50	33.3%
Type of Hospital	Government hospital	113	75.3%
	Non-governmental hospitals	37	24.7%
Age Group	18–24 years old	23	15.3%
	25–34 years old	74	49.4%
	35–44 years old	42	28.0%
	45–54 years old	11	7.3%
Level of education	Diploma	25	16.7%
	Bachelor’s degree	92	61.3%
	Higher education (master or PhD)	33	22.0%
Years in profession	less than 1 years	8	5.3%
	1–5 years	52	34.7%
	6–10 years	37	24.7%
	11–15 years	31	20.7%
	16–20 years	14	9.3%
	> 20 years	8	5.3%
Years in ED setting	less than 1 years	37	24.7%
	1–5 years	70	46.7%
	6–10 years	29	19.3%
	> 10 years	14	9.3%

### Level of perception toward the family presence during resuscitation/CPR in emergency department

This section describes the level of perception toward family presence during resuscitation/CPR in the emergency department. The results indicate that 58% of participants had a good perception, while 42% had a bad perception.

The table summarizes perceptions of family presence during resuscitation/CPR in emergency departments (ED) in Palestine, based on a sample of 150 participants. The overall mean score for the ten items is 2.22 (SD = 0.579), indicating a good perception according to the cut-off point (≥ 2.1 for good perception).

Specific items, however, reveal mixed views. For example, while there is general agreement that staff have adequate knowledge and training (Mean = 2.82) and that dying patients should always have family members present if available (Mean = 2.94), participants expressed bad perceptions regarding the appropriateness of family presence (Mean = 1.85), their comfort level (Mean = 1.82), and the benefits to the patient (Mean = 1.75), the results of nurses’ perceptions are shown in Table 2.

**Table 2:** Perceptions of family presence during resuscitation/CPR in emergency department in Palestine (*n* = 150)

Item	Mean	SD	Status
1. We usually have family members present during resuscitation/CPR in my ED.	2.53	1.39	*Good perception*
2. It is appropriate to have family members present during resuscitation/CRP in the ED.	1.85	1.06	*Bad perception*
3. I feel comfortable having family members present during resuscitation/CPR in the ED.	1.82	1.04	*Bad perception*
4. Nurses and doctors generally agree on whether family members should be present during resuscitation/CPR in the ED.	2.41	1.17	*Good perception*
5. There is enough room in the ED for family members to be present during resuscitation/CPR.	1.91	1.00	*Bad perception*
6. Staff have adequate knowledge and training to support family presence during resuscitation/CPR.	2.82	1.16	*Good perception*
7. Family presence during resuscitation is beneficial for the family.	2.03	1.02	*Bad perception*
8. Family presence during resuscitation is beneficial for the patient.	1.75	0.92	*Bad perception*
9. Families should have unlimited access to the dying patient.	2.22	1.09	*Good perception*
10. Dying patients should always have family members present if they are available.	2.94	1.23	*Good perception*
**Total Mean Score (10 items)**	**2.22**	**0.579**	*Good perception*

Cut of point as follow; <2.1 = bad perception & ≥2.1 = good perception

Median = 2.1

### Differences between demographic variables in terms of perception of total mean score

The analysis compares the perception total mean scores of participants across various demographic variables. Results indicate that sex, workplace, age group, level of education, years in the profession, and years in the ED setting do not show statistically significant differences in perception scores, as all p-values are greater than 0.05.

However, there is a statistically significant difference in perception based on type of hospital (*p* = 0.022), where participants working in non-governmental hospitals (Mean = 2.42, SD = 0.67) scored higher on perception than those in government hospitals (Mean = 2.17, SD = 0.54). This suggests that hospital type may influence perceptions toward family presence during resuscitation/CPR in the emergency department, Table 3 presents the differences between demographic variables in terms of perception of total mean score.

**Table 3:** Differences between demographic variables in terms of perception of total mean score (*n* = 150)

Demographic variables		*n*	Mean	SD	Statistical values	*P*-value
Sex	Male	104	2.22	0.58	T=-0.309 df = 148	0.758
	Female	46	2.25	0.58		
workplace	Northern Palestine	66	2.34	0.59	F = 2.121 df = 2	0.124
	Central Palestine	34	2.16	0.53		
	Southern Palestine	50	2.13	0.58		
Type of Hospital	Government hospital	113	2.17	0.54	T=-2.313 df = 148	0.022*
	Non-governmental hospitals	37	2.42	0.67		
Age Group	18–24 years old	23	2.18	0.50	F = 1.038 df = 3	0.377
	25–34 years old	74	2.28	0.62		
	35–44 years old	42	2.12	0.55		
	45–54 years old	11	2.41	0.51		
Level of education	diploma	25	2.20	0.54	F=0.116 df = 2	0.891
	bachelor’s degree	92	2.22	0.60		
	Higher education (master or PhD)	33	2.27	0.55		
Years in profession	less than 1 years	8	2.16	0.76	F=0.457 df = 5	0.808
	1–5 years	52	2.22	0.61		
	6–10 years	37	2.19	0.54		
	11–15 years	31	2.34	0.48		
	16–20 years	14	2.26	0.79		
	> 20 years	8	2.05	0.24		
Years in ED setting	less than 1 years	37	2.14	0.52	F=0.814 df = 3	0.488
	1–5 years	70	2.30	0.65		
	6–10 years	29	2.20	0.50		
	> 10 years	14	2.14	0.50		

Independent t test and One Way ANOVA tests

*Significant at p = < 0.05

## Discussion

The perspectives of nurses regarding family attendance during CPR in emergency rooms were investigated in this study. Despite recognizing the potential advantages for family members, the results showed that nurses generally had mixed but slightly positive or balanced on family presence during resuscitation. These results demonstrate how difficult it is to strike a balance between clinical effectiveness and family-centered care in emergency settings.

Our findings imply that family members may benefit from family participation during resuscitation; nonetheless, nurses’ opinions remained largely unfavorable. This result is in line found that [8], family members may experience increased emotional distress when resuscitation attempts fail. Observing intrusive operations may exacerbate long-term psychological damage and make grieving more difficult.

On the other hand, new studies have shown that family members can benefit from being present during resuscitation. First, research suggests that allowing family members to participate in resuscitation can increase their sense of involvement in the care process and help them comprehend the efforts of medical professionals, which may lessen feelings of uncertainty and powerlessness during crucial times [7]. Secondly, family members have found their presence to be comforting while witnessing resuscitation efforts, which aligns with family-centered care concepts in promoting emotional closure and a sense of participation [8]. Lastly, family presence has been defined as promoting autonomy, dignity, and emotional support within a care framework that places a high priority on patient-family connections, which is in line with recommendations for modern family-centered practice [9].

Family presence was found to be hampered by elevated stress, perceived workload, and worries about being distracted during CPR [10]. Furthermore, according to recent research, a large number of emergency department healthcare workers are unfamiliar with family-witnessed resuscitation, and the vast majority of them voice concerns about family presence because of worries about interference, elevated stress levels, and challenges juggling clinical duties with family involvement [11]. Additionally, physicians’ opinions regarding family presence are greatly influenced by differences in institutional rules and personal beliefs; healthcare professionals report inconsistent support and advice across settings [12].

Perceptions regarding family attendance during resuscitation seem to be significantly influenced by training and education. It has been shown that structured educational interventions significantly improve nurses’ knowledge and self-confidence in supporting family presence. After nurses completed formal training sessions that involved presentations and simulation videos and guided discussions, the nurses showed greater perceived benefits and self-confidence [13]. Furthermore, comprehensive studies of simulation-based experiences show that simulation can increase therapists’ understanding of this practice and support family presence [14]. Recent studies, however, also show that many nurses working in critical care environments still have low self-confidence and unclear risk-benefit knowledge when it comes to family presence during resuscitation, indicating ongoing deficiencies in training and preparation [15].

In terms of demographic characteristics, the present study found no significant correlation between the perception scores of the nurses and the demographic variables. This is consistent with studies that found that the perception of nurses regarding family presence during resuscitation were not significantly related to demographic variables [16], that found the perceptions of the nurses regarding the presence of the family during the process of resuscitation in the clinical setting were not significantly related to some demographic variables. For example, the cross-sectional study on surgical nurses found that the confidence score and perceptions regarding the presence of the family were not significantly related to the demographic variables such as age, sex, educational attainment, etc [17]. Other studies, however, found that the perceptions and practice of family-centered nursing care might be associated with the demographic characteristics of the nurses. Marital status and interpersonal skills were found to significantly modify the association between the perceptions of the nurses and the implementation of the practice in the aforementioned study on nurses who provided family-centered nursing care to children with chronic illnesses [18]. These findings indicate that the influence of demographic characteristics on perceptions may vary depending on the clinical context and specific aspects of family‑centered care being assessed.

Moreover, some studies suggest that the attitudes and opinions of the staff may not be fully explained by the types of hospital ownership. According to recent research studies, organizational policies and leadership support appear to have a greater influence on staff attitudes than do the investment in nursing services, care quality, and job outcomes among for-profit, non-profit, and governmental hospitals. Such differences do not necessarily lead to significant variations in staff perceptions of their roles and support for family presence in critical care situations [19].

The significant difference observed between governmental and non-governmental hospitals may be attributed to variations in institutional policies, staffing patterns, workload, training opportunities, and organizational culture. Non-governmental hospitals may provide more supportive environments and greater access to training, which could positively influence nurses’ perceptions toward family presence during resuscitation.

Overall, the results of this study emphasize the importance of having formal educational programs, clear institutional policies, and a supportive organizational setting in developing nurses’ attitudes and facilitating the safe practice of family presence during resuscitation in emergency rooms.

### Limitations and strengths

This study has a number of drawbacks. The fact that there are many hospitals in the West Bank means that the time allocated to collect the data was quite short, and the results could have been affected by problems in traveling to and from the hospitals. The fact that the researchers used questionnaires, which were self-administered, could have affected the results, as the nurses could have answered them based on what they thought was socially acceptable rather than what they really did. Despite these limitations, this study has given insightful information about the perceptions of nurses in the Palestinian emergency rooms regarding family presence during cardiopulmonary resuscitation. The fact that the researchers used a multi-center strategy and included nurses from both private and public hospitals made the results more applicable, and this method can help in gaining a wider view of the perceptions about family presence during CPR.

## Conclusion

The perspective of the nurses on family presence during CPR was assessed in the current study. The findings indicated that, although some of the nurses pointed to the emotional support that family members could provide in life-threatening cases, they also pointed to some issues of concern, such as possible disruptions to the procedure and psychological effects on families and medical staff. In order to manage family presence, some of the nurses pointed to the need to have protocols and support mechanisms in place. Overall, the current study indicates that balancing the need to care for patients with the need to care for families is a challenge, as well as a need to conduct more research and come up with better methods to ensure that patient care and family care are met.

### Recommendations and implications

This shows that guidelines, which are evidence-based and consider the patient as well as the family, should be developed to ensure the family is present during CPR. Moreover, the role of healthcare providers should be clearly explained. It is important to note that in order to effectively manage the family presence during CPR, the nurses should receive training regularly. It is important to note that in order to ensure the family is present during CPR in a manner that is consistent with the values of the Palestinians, the policies should consider the culture and religion of the Palestinians. Moreover, the healthcare institutions should ensure the nurses receive psychosocial support, stress management education, and safe environments to voice their concerns and address emotional issues. It is recommended that future studies consider the overall effects of family presence on the patient and the medical personnel, including the family’s views and possible incorporation into CPR decision-making processes. Resource allocation to support effective practice in emergency situations, including the presence of liaison staff and areas to facilitate family interaction without hindering care, can be helpful.

## Data Availability

Data supporting the findings are available from the corresponding author upon reasonable request.

## Abbreviations

CPR: Cardiopulmonary Resuscitation
FBDR: Family presence during resuscitation
